# Design and development of a machine-learning-driven opioid overdose risk prediction tool integrated in electronic health records in primary care settings

**DOI:** 10.1186/s42234-024-00156-3

**Published:** 2024-10-18

**Authors:** Khoa Nguyen, Debbie L. Wilson, Julie Diiulio, Bradley Hall, Laura Militello, Walid F. Gellad, Christopher A. Harle, Motomori Lewis, Siegfried Schmidt, Eric I. Rosenberg, Danielle Nelson, Xing He, Yonghui Wu, Jiang Bian, Stephanie A. S. Staras, Adam J. Gordon, Jerry Cochran, Courtney Kuza, Seonkyeong Yang, Weihsuan Lo-Ciganic

**Affiliations:** 1 Department of Pharmacotherapy and Translational Research, College of Pharmacy, University of Florida, Gainesville, FL USA; 2 Department of Pharmaceutical Outcomes and Policy, College of Pharmacy, University of Florida, Gainesville, FL USA; 3 Applied Decision Science, Cincinnati, OH USA; 4 Division of General Internal Medicine, School of Medicine, University of Pittsburgh, Pittsburgh, PA USA; 5 Center for Pharmaceutical Policy and Prescribing, University of Pittsburgh, Pittsburgh, PA USA; 6 Center for Health Equity Research Promotion, Veterans Affairs Pittsburgh Healthcare System, Pittsburgh, PA USA; 7 Department of Health Policy and Management, Richard M. Fairbanks School of Public Health, Indiana University, Indianapolis, IN USA; 8 Center for Biomedical Informatics, Regenstrief Institute, Indianapolis, IN USA; 9 Department of Community Health and Family Medicine, College of Medicine, University of Florida, Gainesville, FL USA; 10 Division of General Internal Medicine, Department of Medicine, College of Medicine, University of Florida, Gainesville, FL USA; 11 Health Outcomes and Biomedical Informatics, College of Medicine, University of Florida, Gainesville, FL USA; 12 Division of Epidemiology, Department of Internal Medicine, University of Utah, Salt Lake City, UT USA; 13 Informatics, Decision-Enhancement, and Analytic Sciences Center, Veterans Administration Salt Lake City Health Care System, Salt Lake City, UT USA; 14 Geriatric Research Education and Clinical Center, North Florida/South Georgia Veterans Health System, Gainesville, FL USA

**Keywords:** Machine learning, Clinical decision support, User-centered design approach

## Abstract

**Background:**

Integrating advanced machine-learning (ML) algorithms into clinical practice is challenging and requires interdisciplinary collaboration to develop transparent, interpretable, and ethically sound clinical decision support (CDS) tools. We aimed to design a ML-driven CDS tool to predict opioid overdose risk and gather feedback for its integration into the University of Florida Health (UFHealth) electronic health record (EHR) system.

**Methods:**

We used user-centered design methods to integrate the ML algorithm into the EHR system. The backend and UI design sub-teams collaborated closely, both informed by user feedback sessions. We conducted seven user feedback sessions with five UF Health primary care physicians (PCPs) to explore aspects of CDS tools, including workflow, risk display, and risk mitigation strategies. After customizing the tool based on PCPs’ feedback, we held two rounds of one-on-one usability testing sessions with 8 additional PCPs to gather feedback on prototype alerts. These sessions informed iterative UI design and backend processes, including alert frequency and reappearance circumstances.

**Results:**

The backend process development identified needs and requirements from our team, information technology, UFHealth, and PCPs. Thirteen PCPs (male = 62%, White = 85%) participated across 7 user feedback sessions and 8 usability testing sessions. During the user feedback sessions, PCPs (*n* = 5) identified flaws such as the term “high risk” of overdose potentially leading to unintended consequences (e.g., immediate addiction services referrals), offered suggestions, and expressed trust in the tool. In the first usability testing session, PCPs (*n* = 4) emphasized the need for natural risk presentation (e.g., 1 in 200) and suggested displaying the alert multiple times yearly for at-risk patients. Another 4 PCPs in the second usability testing session valued the UFHealth-specific alert for managing new or unfamiliar patients, expressed concerns about PCPs’ workload when prescribing to high-risk patients, and recommended incorporating the details page into training sessions to enhance usability.

**Conclusions:**

The final backend process for our CDS alert aligns with PCP needs and UFHealth standards. Integrating feedback from PCPs in the early development phase of our ML-driven CDS tool helped identify barriers and facilitators in the CDS integration process. This collaborative approach yielded a refined prototype aimed at minimizing unintended consequences and enhancing usability.

**Supplementary Information:**

The online version contains supplementary material available at 10.1186/s42234-024-00156-3.

## Background

The incidence of prescription opioid overdose deaths in the United States (US) surged from 3,442 in 1999 to 16,706 in 2021 (Drug Overdose Death Rates, n.d.). In response, healthcare systems, payers, and policymakers have launched various programs to identify and manage patients at high risk of overdose and opioid use disorder (OUD). These initiatives include prior authorizations to prevent potentially inappropriate opioid prescriptions, referrals to addiction or pain management specialists, and enrollment in prescription lock-in programs (Roberts et al. 2016; Academies and of Sciences E, 2017). Yet, these interventions can be costly and burdensome for patients, clinicians, and payors.

Accurately identifying patients at high risk of overdose and OUD may help combat the opioid crisis. However, traditional risk-factor-based measures or interventions often miss 70%-80% of individuals with overdose or OUD (Wei et al. 2019; Lo-Ciganic et al. 2019, 2022). Machine learning (ML) and artificial intelligence (AI) algorithms offer promising alternatives, using real-world healthcare data to improve prediction accuracy and more efficiently identify those at highest risk for targeted interventions. Our prior research has developed and validated ML algorithms for predicting opioid overdose and OUD across different populations (e.g., Medicare, Medicaid) and different data sources (e.g., claims data and integrated human services and criminal justice data) (Lo-Ciganic et al. 2019, 2022, 2020, 2021). These ML algorithms have proven more accurate at identifying high-risk individuals than current risk measures (Lo-Ciganic et al. 2019, 2022).

Building on our foundational work, the goal of this study was to develop an ML-driven clinical decision support (CDS) tool to aid primary care clinicians in identifying patients at elevated opioid overdose risk. Despite advancements in developing ML algorithms for disease detection and diagnosis, challenges persist in implementing these models in routine clinical practice. For example, data quality concerns can lead to biased models and inaccurate predictions and can exacerbate health disparities (Obermeyer et al. 2019; Wiens et al. 2019). Also, the lack of interpretability in some ML models (e.g., deep learning) may hinder clinician trust and adoption (Teng et al. 2022; Vellido 2020; Gomolin et al. 2020). Furthermore, the design, development, and implementation of ML and AI-driven CDS tools with seamless integration into clinical workflows poses additional hurdles (e.g., complex algorithm integration and update frequency). Ethical and legal concerns regarding patient privacy and liability must be addressed, alongside rigorous validation and evaluation of these tools' effectiveness in real-world settings (Wiens et al. 2019). Addressing these challenges requires interdisciplinary collaboration to develop transparent, interpretable, and ethically sound ML-driven CDS solutions that prioritize patient safety and privacy while seamlessly integrating into clinical practice.

To enhance guidance on the design, development, and implementation of such a CDS tool, we established a backend process that happens behind the scenes in a software system for integrating our ML-based ML algorithm, which predicts opioid overdose risk, into the electronic health records (EHR) system. Additionally, we adopted a user-centered design (UCD) approach to develop the frontend interface that users interact with directly of the ML-driven CDS tool at University of Florida Health (UF Health). This CDS tool is specifically designed to identify patients at elevated risk for opioid overdose within the next three months at the time of prescribing an opioid, targeting those seen at UF Health internal medicine and family medicine clinics.

## Methods

We followed a UCD framework. UCD is often referred to in a broad sense as “the active involvement of users for a clear understanding of user and task requirements, iterative design and evaluation, and a multi-disciplinary approach” (Mao et al. 2005). UCD methods were initially developed in the context of commercial product design(Norman and Draper 1986) and have since been expanded and refined to address user needs in a broad range of contexts (Vredenburg et al. 2002; Abras 2004). The recent proliferation of ML/AI-driven CDS tools underscores the need for adapting UCD methods to ensure these tools effectively support clinical work as intended. Supplemental Fig. 1 provides an overview of the UCD activities used in the development of our CDS tool. The study was approved by the UF Institutional Review Board (IRB202002225) and was funded by the National Institute on Drug Abuse (R01DA050676).

### Backend process: ML algorithm integration

In separate work not reported on here, we developed a ML algorithm for predicting opioid overdose using data from the OneFlorida + Clinical Research Network (OneFlorida Clinical Research Consortium. 2021; Hogan et al. 2022), a Patient-Centered Outcomes Research Network (PCORnet) (Fleurence et al. 2014). This data included 2017-2022 EHR data covering > 50% of Floridians across 22 hospitals. Subsequently, we validated and refined the algorithm using data from the UF Health population. The integration process development initiated with a review of published clinical workflows (Price-Haywood et al. 2018; Knox and C. 2015; Hudson and Wimsatt 2014; Hans et al. 2018; Sutton et al. 2020). Subsequently, our team created a preliminary plan (Supplemental Fig. 2) using Microsoft Visio (Redmond, WA, USA) to illustrate the data flow from the EHR for applying the algorithm and the transmission of risk scores to the patient charts. We held meetings with members of UF Health information technology (IT) and Integrated Data Repository (IDR) Research Services to understand the existing process for alert implementation and discuss the flow of data, server specifications, software, hardware, costs, and determine responsibilities (e.g., data access, code development). The UF Health IDR, supported by the UF Clinical and Translational Science Institute (CTSI), serves as a comprehensive database that collects and organizes information from across UF Health’s clinical and research endeavors (Integrated Data Repository Research Services n.d.). Risk assessments were also conducted with members of UF and UF Health IT staff in order to obtain permission to pull patient health information (PHI) for the risk score development and to build the CDS in the clinical EHR system. First, we obtained patient PHI data from IDR for patients aged ≥ 18 years with a record of an opioid medication from an outpatient UF Health Internal Medicine or Family Medicine clinic in Gainesville, FL in the prior year to generate risk scores. Specifically, we used Python to create predictors and applied the developed algorithm to generate risk scores. The process runs on an internal UF Health server to ensure PHI safety. Second, the generated risk scores are then integrated into Epic® EHR system. UF Health adopted the Epic® EHR in 2011 and employs it in inpatient and outpatient settings.

Initially, we proposed several use cases and triggering criteria for integrating alerts into the clinician workflow. One proposed use case was to flag a patient during order entry of an opioid prescription. The initial criteria for triggering an alert included: (1) the patient was identified as high risk by the ML algorithm; (2) naloxone was not included in the current prescription order and had not been ordered in the past year; and (3) the clinician had not seen an opioid overdose alert for that patient in the past six months. A second use case involved providing a nurse manager with a weekly list of high-risk individuals at the clinic level. A third use case was a passive alert that displayed on the patient’s storyboard.

### Frontend User Interface (UI) development

**Fig. 1:**
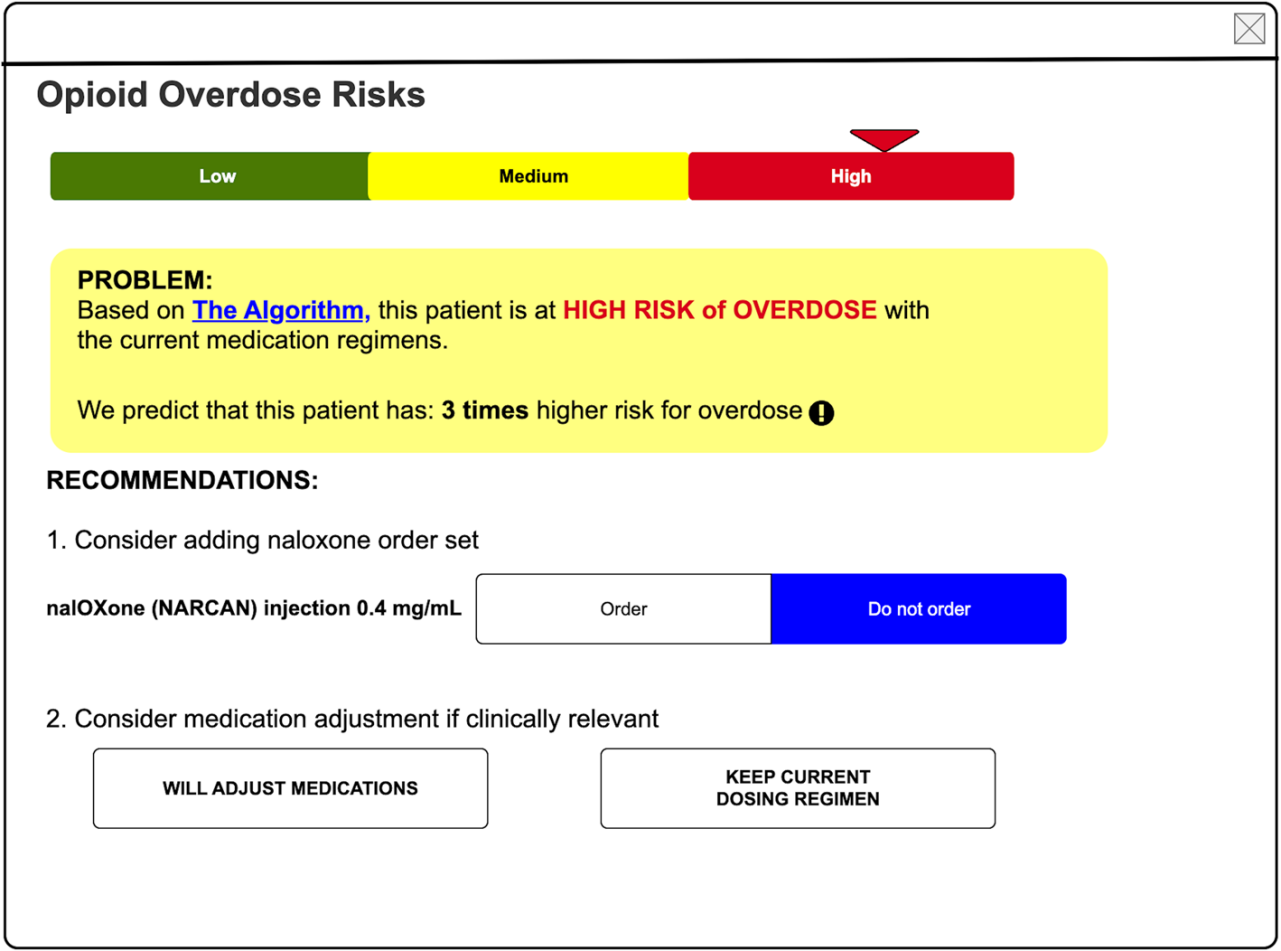
Before end-user input: an illustration of a proposed opioid overdose risk prediction clinical decision support tool

With regard to frontend user interface (UI) development, our research investigator (WL) drafted an initial UI design concept (Fig. 1) based on the literature and our study goals prior to user feedback sessions. The CDS was built by two UF Informatics pharmacists (KN, BH).

#### Study participants

User feedback sessions included PCPs from UF Health family medicine and internal medicine groups with self-reported experience in prescribing opioids. PCPs were recruited to participate in either formative interviews or usability sessions through department grand rounds presentations and emails. A waiver of documentation of consent was provided, and participants were required to agree before initiating a Research Electronic Data Capture (RedCap; Nashville, TN, USA) questionnaire designed to collect demographic information. All user feedback sessions, hosted on Zoom (San Jose, CA, USA), were recorded and transcribed, with each session lasting approximately 60 min.

#### Formative interviews

We conducted a series of formative interviews to guide the development of the frontend UI. Given the limited access to PCPs due to their busy schedules, we assembled a panel of 5 experienced PCPs to provide feedback and designed 3 relatively short formative interview sessions, each lasting approximately 60 min. All panelists were invited to each session. Having a pool of panelists willing to participate reduced recruitment time and allowed for the development team to obtain input from users when it was most needed without significant disruption to development schedules.

For the formative interview sessions, we created interview guides inspired by the CDS five rights framework (Campbell 2013). Each formative interview session emphasized one of the 3 key topics, and we tailored questions to address the right information, right person, right format, right channel, and right time in the workflow. Semi-structured interviews were facilitated by a human factors expert, with a notetaker and an observer present. A 30-min debriefing session followed each interview to share key insights among the facilitator, notetaker, and observer. All PCP participants received a $50 gift card after each session as a token of appreciation for their involvement in the study.

Findings from the formative interviews led to a set of UI design recommendations. The backend and frontend sub-teams held a series of design meetings to discuss feasibility of specific UI design recommendations and adapted as appropriate.

#### Usability sessions

To obtain feedback about the usability of the alert, we recruited PCPs who had not previously participated in formative interviews. Using Axure (San Diego, CA, USA), a human factors visual designer (JD) built a clickable prototype based on a version originally built by informatics pharmacists (KN and BH). Participants accessed the prototype via a web browser and shared their screens through Zoom. They were asked to recall a recent case involving an opioid prescription order for a patient and to then imagine the alert appeared when they signed the order. As they interacted with the prototype, they were prompted to share their thoughts (i.e., a “”think aloud” protocol) (Boren and Ramey 2000). Four one-on-one usability sessions were conducted and analyzed between March 27, 2023 and April 10, 2023 using the same process as used in the formative interviews. After each usability session, the facilitator, notetaker, and observers took part in a debrief to discuss insights. Refinements to the UI were presented in the redesign and a second round of 4 one-on-one usability sessions was conducted between June 6, 2023 and June 13, 2023 using the same methods and process described above. This feedback was then integrated into the design.

### Data analysis

For the formative interviews, notes from all observers were integrated into a single document. One investigator (JD) reviewed the notes and extracted themes and insights with implications for design. For each theme, the investigator included related quotes and design recommendations. Themes, quotes, and design recommendations were recorded in PowerPoint slides and shared with the project team for additional discussion and interpretation.

For the usability sessions, one investigator (JD) reviewed debrief notes and more in-depth notes from usability sessions to extract themes and insights. As a validity check, a second investigator (DW) reviewed transcripts and found quotes supporting each insight.

## Results

### Backend process: ML algorithm integration

**Fig. 2:**
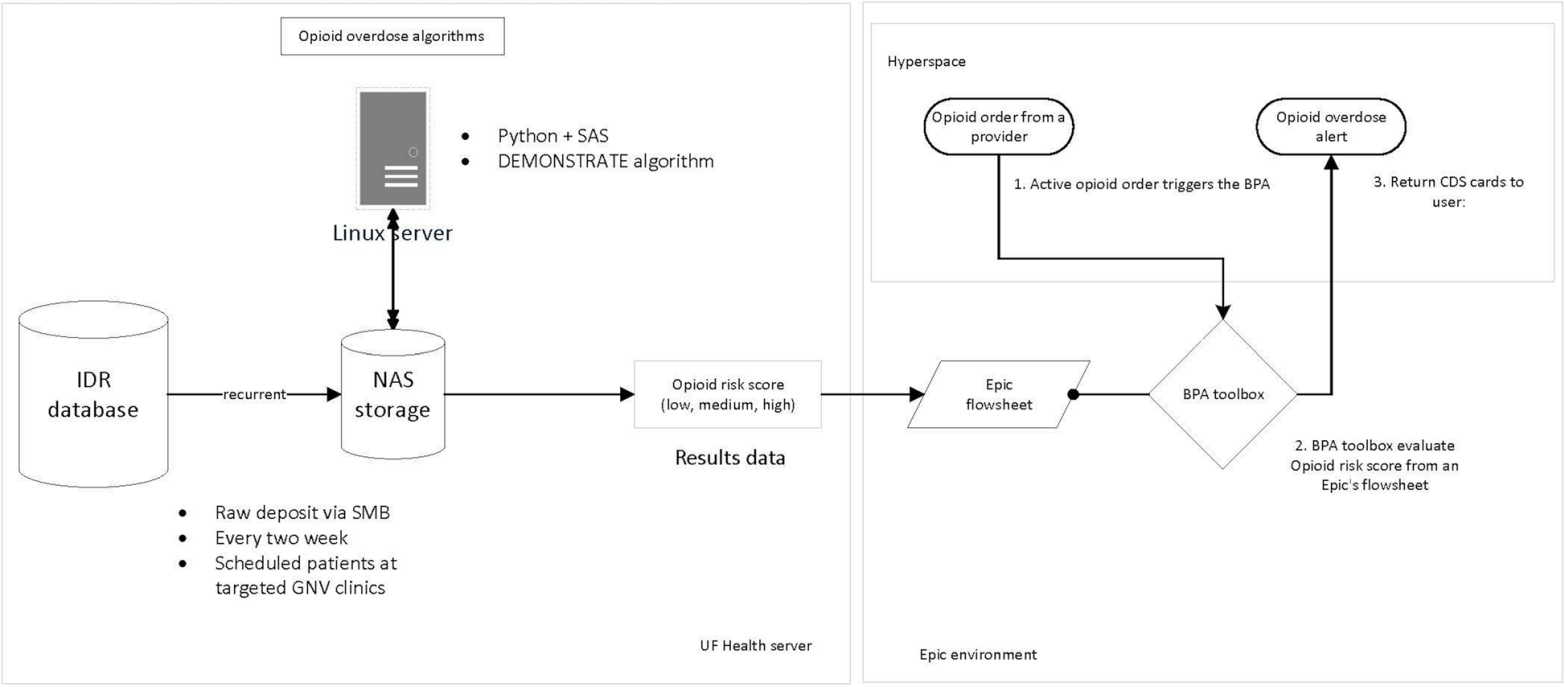
After input from end-users*,* UF Health information technology*,* and Integrated Data Repository Research Services: An illustration of the adopted workflow diagram for the opioid overdose risk prediction clinical decision support tool

Figure 2 presents the final design of our ML algorithm integration process. Our ML algorithm operates on a local UF Health server, generating risk scores biweekly using recurrent data from the UF IDR database. The eligible cohort, selected from the IDR database, comprises patients aged ≥ 18 years who had at least one outpatient visit at a UF Health clinic and an outpatient opioid prescription within the prior year, and had upcoming appointments at UF Health outpatient clinics (family medicine or internal medicine in Gainesville, FL) within the next two weeks. This ensures timely availability of risk scores and appropriate alert generation during clinic visits. The generated risk scores with risk subgroup categories (i.e., top 5th percentile, top 6th-10th percentile, decile 2, decile 3,… and decile 10) are stored securely in the UF Health server. The risk score is integrated into the Epic electronic health record system using the HL7 standard, appearing as a flowsheet value. This encounter-specific flowsheet is not directly accessible to either healthcare providers or patients, ensuring controlled dissemination of the risk assessment information.

Our design involves integrating the ML opioid overdose risk score into the Epic® EHR environment using Health Level 7 standards with an application programming interface (API). Based on the feedback from formative interviews (details see below section), the final ML-driven CDS will interruptedly trigger in the form of a best practice alert (BPA) when a clinician attempts to sign an opioid prescription order if: (1) the algorithm has determined the patient is at elevated risk (about 1 out of 5000 patients); (2) naloxone is not part of the current prescription order set, nor has naloxone been ordered in the past year; and (3) the clinician has not seen the opioid overdose alert for that patient in over one year, based on the interview results described below.

The UCD feedback led to a significant revision of our alert system. We shifted from passive alerts on the storyboard to an interruptive alert that fires when the prescriber signs the order. This change addresses several concerns raised by PCPs: (1) Passive alerts became less effective over time; (2) They were redundant with the Prescription Drug Monitoring Program (PDMP); (3) They added unnecessary clutter to the interface, and (4) They were time-consuming to address. The new alert timing (at signing order instead of placing order) ensures that PCPs don't miss notifications when support staff preload orders. Additionally, the alert now fires for each prescriber per patient, resolving concerns about previous triggers for other providers. To mitigate alert fatigue, we've extended the alert firing frequency from six to 12 months, reducing the volume of non-relevant alerts. These modifications have shaped our final alert triggering criteria, which are as follows: (1) patient categorized as high risk based on our machine learning model, (2) naloxone was not included in the current prescription order, and (3) the same alert was not fired for the last 12 months.

The final ML-driven CDS tool for opioid overdose will replace an existing opioid-induced respiratory depression (OIRD) BPA when the OIRD BPA and ML-driven CDS BPA criteria overlap. If a patient’s case meets the existing ORID BPA alert but not our ML-algorithm criteria, the existing ORID alert will still trigger.

### Frontend UI development

#### Results from the formative interviews

We conducted 7 formative interviews including 3 related to workflow process (2 individual interviews; one dyad interview), 2 related to displaying risk (2 dyad interviews), and 2 related to risk mitigation strategies (1 individual interview, 1 dyad interview). Table 1 presents example quotes from the formative interviews.

**Table 1:** Example Quotes by Domain from the expert-user formative interviews

**Alerts sessions** (*n* = 4 PCPs; 2 one-on-one interviews and 1 dyad interview)	**Displaying risk sessions** (*n* = 4 PCPs; 2 dyad interviews)	**Risk mitigation strategies sessions** (*n* = 3 PCPs; 1 one-on-one interview and 1 dyad interview)
**• Similarity of the CDS opioid overdose tool to the existing alert for OIRD:** *“When I look at this…both of these are trying to alert me to risk of overdose and do I want to try a different medicine. If this is actually doing things better and taking into account more things, maybe if the alert would reflect that. That way it may catch the attention of more people.”*	**• Risk descriptions:** *“10 times higher is an impressive number…if you want me to act on something, it needs to be convincing”* *“higher than what?”* **• Low value of graphical risk representations:** *“Boils down to this person is at high risk and I need to act on it. That is all I need to know.”*	**• Comments for naloxone options in the existing OIRD alert:** *“Intranasal is fine; [it’s] usually the cheapest one. Evzio is very expensive. None of the insurance companies cover that. They cover a generic solution for that. Whoever created this didn’t look at the prices.”* *“The other thing is you limit yourself to injections, but what you want is nasal spray. That’s the cheap one you want…. If you only have this as an order, I would not order it here, because you’ve got the wrong medicine there…. Most people don’t like to give injections, especially to someone else. You tell someone to spray it up the nose, a kid can do that…. Most insurance companies cover nasal spray.”*
**• High-volume of non-relevant alerts:** *“Even for drug-drug interactions, we have only serious alerts turned on. If you have moderate to mild turned on, too many alerts, no one reads anything.”* *“So much is unimportant that I have to override, so I worry that I override something important."*	**• Timeframe of 3-month risk:** *“This shows how severe this is, and it quantifies it. It is more information that impresses risk.”* *“I don’t know how to make sense of this…2% chance of overdose in 3 months sounds bad.”* *“I don’t think this would change my practice at all.”*	**• Concerns for naloxone order**: *“I will try to find a way to reduce the risk so that I won’t have to prescribe it.”* *“My experience is that most of the patients, or some of them, decline the medication.”* “S*ome of them, previously when I prescribed it… they've come back and I've asked them about it, they didn't pick it up.”* *“Some refuse to get it.”* *“You should have three buttons: order, do not order, and patient declined.”*
**• Repetitive alerts for patients on long term therapy who have tolerated it well and the PCP knows them well** *“In my case, the vast majority are refills, so I don’t actually benefit from the [OIRD tool]. I can’t really remember if I read it the first or second time I saw it.”*	**• Regarding risk for opioid overdose and risk of developing opioid use disorder:** *“Clinically, I see these going hand in hand. If not, I want to know why.”*	**• Specialist referral/consultation strategy:** *“That’s a no-brainer. I tell residents to refer if uncertain. You have to grow a strong back. This is such a dangerous drug; you can’t allow people to do what they want.”* *“If you are thinking OUD, you should basically adjust, come off – there would be the specialist referral, consultation. You would have to address that.”* *“I’d be more likely to say ‘We're not going to increase your dose today. I’m going to refer you to a pain management specialist.’”*
**• Passive alerts that sit in the storyboard area:** *“[I] prescribed to a patient the other day and it took 1.5 h to set up contract and complete the paperwork…[I] Will not act on [a passive alert]. Too involved of a process.”*	**• Predictors and trust:** *“I didn’t realize you had 118 things behind it. That boosted it up for me.”* *“Something in me says more is better. A lot of data, that’s got to be good.”* *“I don’t know if any of these [predictors] are modifiable; they won’t help me make them lower risk.”* *“Would [these predictors] help me do anything differently? I think no.”* *“Predictors might come into discussion for why I am not prescribing for them.”*	**• Dose adjustment strategy:** *“I would hope that if you prescribe these medications that you know how to adjust dosages. We actually teach that in the residency program. However, if you have been out there and have not had any pain management training in the past, you might need help.”* *“Just because someone is on an opioid doesn't mean they have to continue for the rest of their life.”*
**• Desire to see the predictors that drive the patient’s risk*:*** *“That will be really important. If it’s just age and everything else was OK, I might still be comfortable…If it was something I wasn’t looking at, it would be very helpful.”* *“[I] would pay more attention if I knew.”*	**• Algorithm validity and trust:** *“I love the idea, but you have to validate this stuff.”* *“Unless the tool is somehow validated in patients, I wouldn’t trust [it].”*	**• Adjuvant therapy strategy:** *“Before prescribing opioids, you have to discuss nonpharmacological and adjuvant type therapy.”* *“Alternative pain management options, that's actually on every prescription, and we already have to click that we've discussed or tried gabapentin or something like that.”*
**• Recommendation for forceful language for naloxone:** *“When it comes to recommendations… what has been happening is that CDC stated that any opioid can kill. I do a lot of medical-legal work. In the climate we are practicing in, if you have anybody on a chronic dose of opioid, you should have them on Narcan period. Make this recommendation more forceful; don’t just [say] ‘consider Narcan’.”*	**• Expectation to change treatment plans or refer or dismiss the patient based on the alert:** *“When high risk comes up, that should be a strong indicator… that I am not going to give you this; I am going to send you to a specialist.”* *“The decision process is really, if that patient is high risk, I would send them to [an] addiction counselor, unless they have cancer.”* *“I would not treat a patient with high overdose risk any differently than a patient with high risk for OUD. Both are the same in clinical practice and would be referred to pain management clinic.”*	**• Screening strategy:** *“You have to check—every time—the PDMP and the urine drug screens, according to the law.”* *“I use the Opioid Risk Tool, and I also have them sign a pain contract.”* **• Patient Education Strategy:** *“I always educate the patients.”*

*Abbreviations*: *CDC* Center for Disease Control and Prevention, *CDS* clinical decision support, *OIRD* opioid-induced respiratory depression, *OUD* opioid use disorder, *PCP* primary care physician, *PDMP* Prescription Drug Monitoring Program, *UF* University of Florida

During the alerts formative interview sessions (*n* = 4 PCPs), it became evident that PCPs found it challenging to differentiate the opioid overdose CDS tool from the existing OIRD alert. They expressed reluctance towards multiple alerts of similar nature during the ordering process and emphasized the importance of understanding why a patient was identified as high risk for overdose.

In the displaying risk formative interview sessions (*n* = 4 PCPs), following a review of the 6 options for presenting risk (Supplemental Table 1) provided by the research team, PCPs emphasized the need to understand the severity of risk and suggested incorporating a 3-month timeframe to accurately quantify risk. They stressed the importance of minimizing false positives to avoid alert fatigue. Additionally, PCPs reported their use of existing tools such as the PDMP, Opioid Risk Tool (ORT), Patient Health Questionnaire (PHQ) 9, Generalized Anxiety Disorder Assessment (GAD-7), Mood Disorder Questionnaire (MDQ), and urine drug screening for opioid overdose and use disorder risk assessment.

The PCPs expressed confidence and trust in our ML-driven CDS tool for risk assessment and decision-making, provided that our algorithm was validated. They also indicated interest in receiving alerts for opioid overdose and OUD; however, any reporting inconsistencies between the two alerts would decrease their trust in the tool. While showing predictors selected in the ML algorithm improved PCP’s trust in the alert, they found the information excessive and suggested including a comprehensive list on a more details tab. The risk formative interview sessions also revealed an unforeseen or unintended outcome of the alert, wherein patients were immediately referred to pain management or addiction/mental health specialists instead of receiving careful evaluation and close follow-up care for opioid use within primary care.

In the risk mitigation strategies formative interview sessions (*n* = 3 PCPs), PCPs favored including up to three risk mitigation options for the alert. They ranked the risk mitigation strategies as follows: (1) opioid dose adjustment, (2) minimizing concurrent high-risk medication use (e.g., benzodiazepines), (3) specialist referral, and (4) prescribing naloxone. They also suggested including alternative risk reduction approaches beyond prescribing naloxone. PCPs identified interface flaws and suggestions, such as simplifying the display, providing additional resources and materials accessible through a details tab or link, integrating acknowledgment options, and preferring nasal spray over injection for naloxone prescription due to its ease of use. Furthermore, PCPs expressed that it may not be necessary to include the use of the PDMP and ordering urine drug screening in the CDS tool, as these are required measures.

PCPs offered detailed feedback on the alert’s appearance, advocating for the judicious use of color, minimizing text, avoiding all-capital text, and placing close/dismiss buttons on bottom right to maintain consistency with other alerts. They also recommended incorporating acknowledgment options such as “patient has naloxone”, “patient declined”, and “other/comment”.

**Fig. 3:**
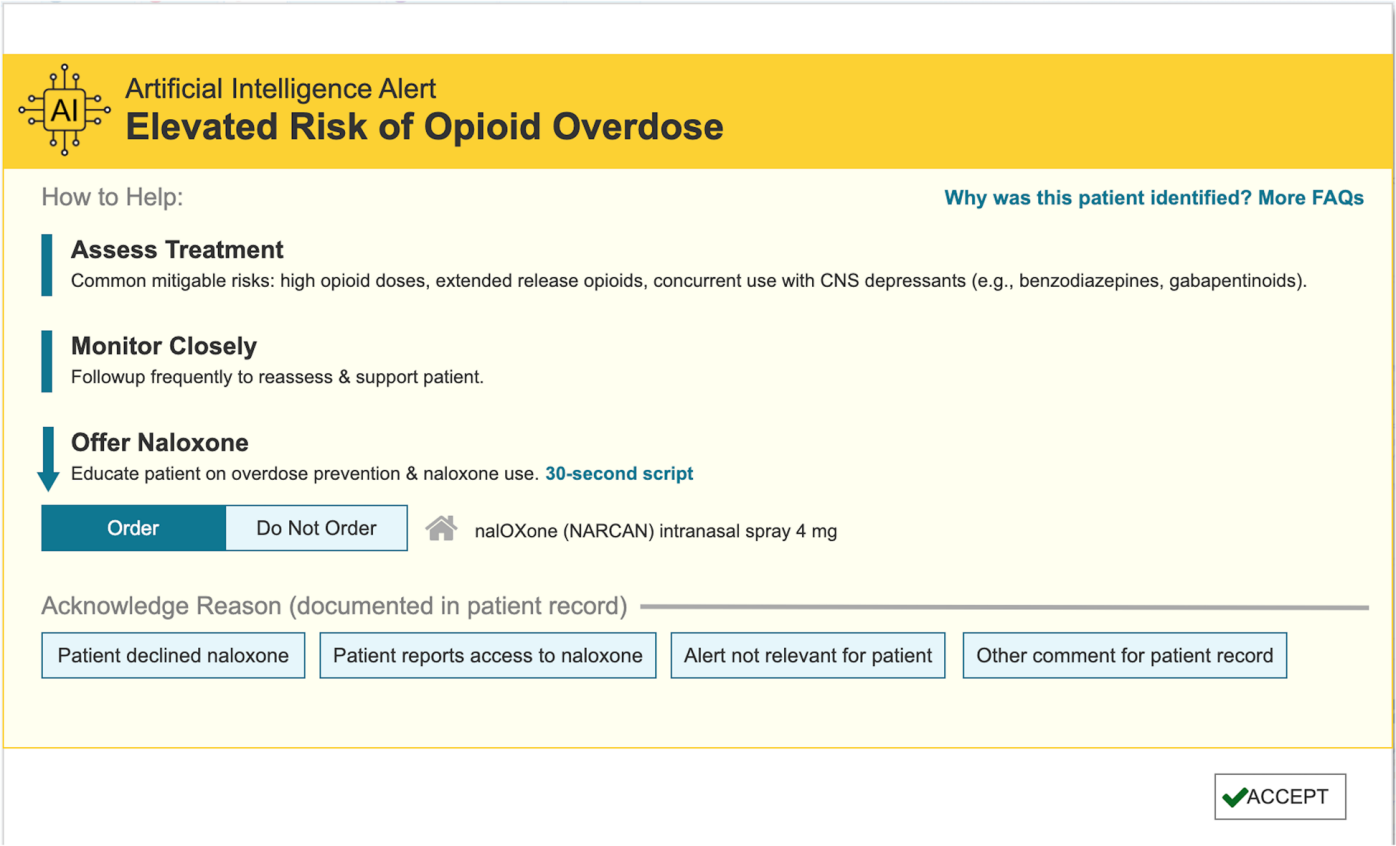
User interface Design 1.0: A revised opioid overdose risk prediction clinical decision support tool after formative interviews

Figure 3 presents our alert (UI Design 1.0) redesigned incorporating feedback from the formative interview sessions. Based on the feedback, we also created concise scripts and developed a frequently asked questions (FAQ) page. Given concerns about only referring patients to specialists or abrupt opioid discontinuation, the redesign aimed to transparently present the risk (i.e., elevated risk of opioid overdose), provide PCPs with minimal necessary information to inform their clinical decision making and improve patient care.

#### Usability Testing Sessions

The two waves each with 4 one-on-one usability testing sessions involving PCPs who had not participated in previous interviews. In the first wave of usability testing sessions, PCP participants did not initially notice that the alert was ML/AI-driven. Most (*n* = 3) ordered naloxone during the usability session. None said they would dismiss the patient or suddenly deprescribe for an existing patient, but they would re-consider prescribing if the alert appeared when seeing new patients. Because the alert was different than the existing naloxone alert, three said they would look at it more closely. They were not in agreement on the timing of the interval for re-appearance if they did not order naloxone. They appreciated the timing of the alert and found it helpful for discussing naloxone with patients. However, some were uncertain about the recommendation to monitor patients closely due to its lack of specificity. After reviewing the FAQ document, all participants understood the alert’s purpose, though some expressed concerns about time constraints limiting their ability to review the FAQ during the clinic visit (but felt it could be used for training residents). The FAQ risk statement (1/125) made PCP participants feel that they should pay attention to the alert and yet did not alarm them to the point of dismissing the patient or suddenly stop prescribing. Also, the list of predictors was considered too “busy” and some were considered unclear or nebulous.

Following the first wave of usability sessions, we made several adjustments based on feedback, including updating the FAQ, moving the risk statement (1/125) from the FAQ to the main alert page, reducing the text in the CDS and FAQ, improving the visibility of the links, updating the override reasons, revising the re-appearance interval to 6 months and clarifying that the algorithm does not use PDMP data in UI Design 2.0.

In the second wave of usability testing, one participant immediately recognized that the alert was driven by AI. Some participants noted the alert’s novel design, which prompted closer attention. They appreciated its advantages over the existing alert, particularly its tailored approach to UF Health. For example, one PCP said “We're clearly looking at multiple predictors specific to UF. So, this wasn't some risk calculator tool developed in some other part of the country that doesn't necessarily apply to our community. It's been developed specifically for our system. I think that … is very valuable.” They found it effective for reminding them to order naloxone and agreed that the timing of the alert’s appearance at the point of ordering was appropriate and agreed on the appearance intervals, such as a 6-month re-appearance interval if naloxone was ordered. They believed that the alert would be beneficial for new patients or unfamiliar patients, including those of colleagues. Overall, PCPs found the override reasons appropriate and comprehensive. Some expressed concerns about potential burden for physicians who frequently prescribe to high-risk patients. Some confusion remained regarding overriding reasons and suggested actions. For instance, a participant selected ‘order’, and then selected ‘will review/discuss with patient’. The participant planned to do both activities and did not expect the toggle to automatically switch to ‘do not order’ when they selected ‘will review/discuss with patient’.

**Fig. 4:**
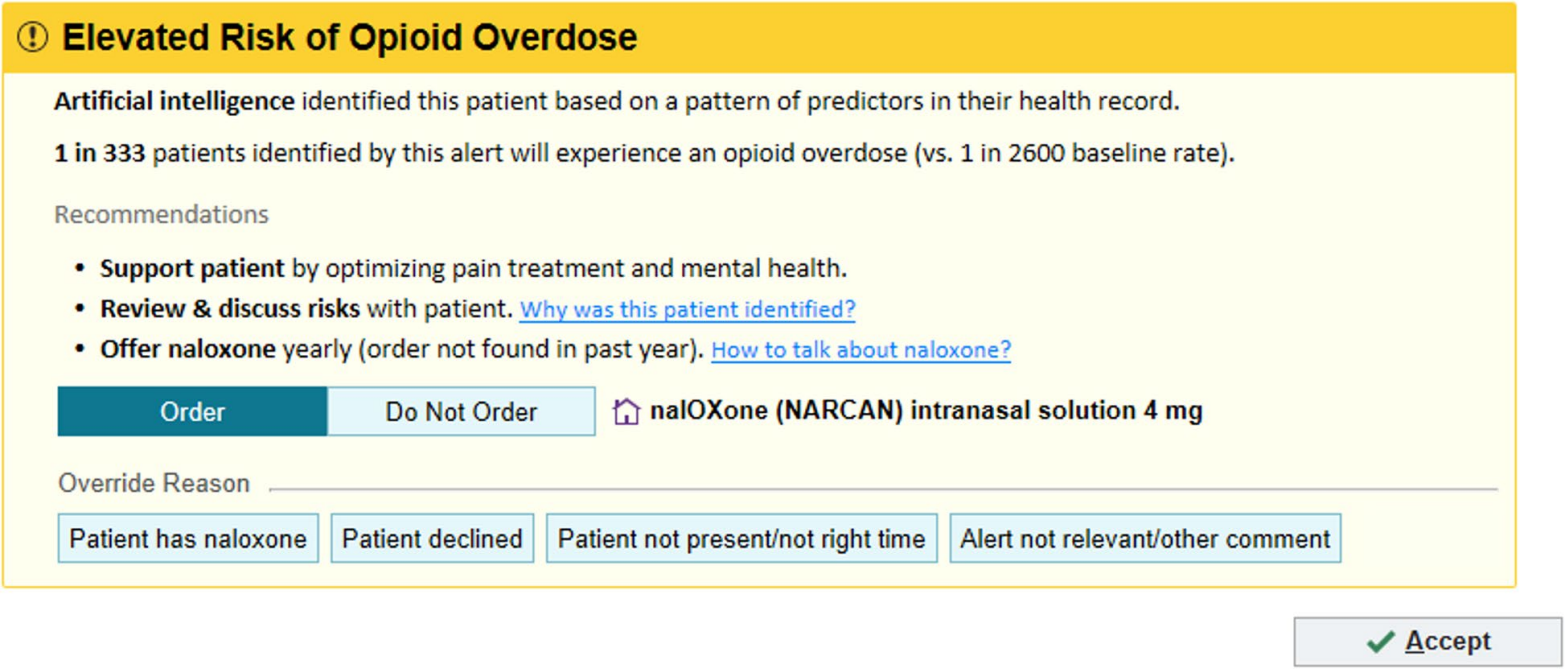
User Interface Design 3.0*: The final opioid overdose risk prediction clinical decision support tool after usability sessions * © 2024 Epic Systems Corporation

While the FAQ page was praised for its educational value and transparency, PCPs indicated they would only click on it initially or when time permitted. They suggested incorporating it into CDS or BPA training before release to avoid surprises. Based on the second wave of usability sessions, we made final modifications to the CDS, including refining recommended actions, rewording and reordering override reasons, updating the FAQ document and scripts, and planning training sessions for end users. Figure 4 depicts our final CDS tool (UI Design 3.0).

## Discussion

In this study, building upon our prior work that created ML algorithms for predicting OUD (Lo-Ciganic et al. 2020) and opioid overdose (Lo-Ciganic et al. 2019), we further tailored a ML-driven CDS tool for predicting opioid overdose and an implementation process specifically designed for primary care internal medicine and family medicine clinics within a university health system. We identified significant challenges in transitioning a validated ML algorithm into a practical CDS tool and integrating this tool in real-world EHRs and healthcare workflow. To our knowledge, this marks the first translation of a ML algorithm predicting opioid overdose into a CDS using a comprehensive UCD approach. This ML-driven opioid overdose CDS tool aims to aid clinicians in making informed decisions at the point of ordering an opioid prescription.

Currently, there is an increasing interest in applying ML-driven CDS tools to aid health professionals in decision-making processes such as psychological resilience of women undergoing treatment for breast cancer (Cm et al. 2023), severe sepsis and septic shock (Giannini et al. 2019; Joshi et al. 2022; Adams et al. 2022), and epilepsy surgical candidate identification (Kanbar et al. 2022). These tools leverage vast amounts of patient data (e.g., insurance claims or EHR data) to provide personalized recommendations and predictions, enhancing clinical outcomes and patient care. In response to the opioid crisis in the US, the growing research on ML algorithms to predict OUD (Garbin et al. 2023) and overdose (Lo-Ciganic et al. 2019, 2022, 2021; Dong et al. 2019, 2021; Ripperger et al. 2021; Gellad et al. 2023; Sun et al. 2020) risk in the US has focused on utilizing data from sources like claims, EHR, and PDMP databases. ML-based prediction tools surpass existing tools, including drug screens, PDMP, ORT, PHQ-9, GAD-7, and MDQ. Although many predictive models have been developed, few have been integrated into CDS tools with careful validation for healthcare professionals (Minegishi et al. 2022; Huizenga et al. 2016; hc1 Opioid Advisor. n.d.; Epic. 2019; Meadows et al. 2018).

Implementing ML-driven tools for predicting opioid overdose is challenging due to data imbalance, with rare events like overdoses creating datasets skewed towards non-occurrences (Cartus et al. 2023). Limited data on rare occurrences like overdoses complicates model training (Cartus et al. 2023) and requires techniques such as oversampling (Johnson and Khoshgoftaar 2019). Additionally, these models must account for dynamic and complex factors, some of which might be difficult to quantify or collect including the evolving nature of opioid types involved in overdoses (i.e., prescription opioids to heroin then fentanyl). Selecting the threshold to identify high-risk individuals requires careful consideration to minimize false positives and alert fatigue, balancing resources available for intervention, and considering the type of intervention and potential unintended consequences. Our study underscores that transparency in ML-driven CDS tools is crucial to increase trust in their usage. While it is essential to perform bias assessments during the model development phase, ethical and bias considerations should be also assessed during the frontend CDS tool development phase to prevent incorrect interpretations or unintended impacts on patients, PCPs, and communities. Our study emphasizes the need for careful development including temporal and external validation (Habib et al. 2021), deployment, and interpretation of these ML-driven models.

Our ML-driven CDS tool for predicting opioid overdose offers valuable insights for future AI-based CDS tools in healthcare. First, the design process must work within the limitations of existing EHR systems and policies within healthcare systems. Iterative user-feedback sessions, crucial for understanding clinicians’ interpretations of ML outputs, help shape an interface that reduces misconceptions and bias (Lowry et al. 2012; Stanton et al. 2017). Tools like wireframes and scenarios are essential in these sessions, despite the challenges in scheduling user sessions. Second, the CDS tool must seamlessly integrate into end users’ decision-making processes and workflows. Involving end users (i.e. PCPs in our study) in the design and evaluation process ensures that the tool complements their decision-making, enhancing acceptance and utilization. While multiple options (e.g. sequential nudges at different points) have been explored and evaluated, we believe a CDS tool with minimal interruption is the most appropriate and effective option that can integrate with PCPs’ workflows. Third, a key aspect is addressing how UIs can unintentionally exacerbate bias (Buonora et al. 2024), as evidenced by an example from opioid overdose risk prediction. An initial design in our opioid overdose risk tool using a red pointer to indicate high risk led to misinterpretations, prompting redesigns to use terms like "elevated risk" and making recommended actions more salient than risk statements. Communicating risk effectively is crucial to avoid biases and errors. For instance, presenting risk as frequency statements (e.g., 1 in 125) is clearer than probabilities (e.g., 2%) or risk categories (e.g., high risk), aiding clinicians in making informed decisions and avoiding bias. Finally, we plan to use a blend of new intraoperative technologies and standards (like HL7 and FHIR) with existing engines from EHR systems (like EPIC®) to create a CDS tool that is both innovative and familiar to PCPs.

ML algorithms developed using multiple large datasets from various and diverse centers and systems tend to be more generalizable (Rockenschaub et al. 2024; Gong et al. 2023). Therefore, our initial ML algorithm for predicting opioid overdose was developed using data from a Patient-Centered Outcomes Research Network (PCORnet) (Fleurence et al. 2014), the OneFlorida + Clinical Research Network (OneFlorida Clinical Research Consortium. 2021; Hogan et al. 2022), which included EHR data (2017-2022) covering > 50% Floridians across 22 Florida hospitals. We then validated and refined the algorithm in the UF Health population. Additionally, we used the UCD approach to develop the frontend and backend implementation processes that can be replicable across other sites and systems. While adopting an ML-based tool with proven validity and reliability—such as ours—eliminates the need for other UF Health sites or other healthcare systems to develop their own ML algorithm, it is advisable for these sites to validate the tool’s accuracy within their specific populations and tailor it to their clinical and operational needs before implementation. This approach could enhance adoption by end users, as our UCD interviews showed that PCPs had increased trust because the tool was validated in the UF Health system’s patient population and included processes designed to meet the sites’ and end-users’ specific needs.

Adaption of the tool to additional sites within UF Health or to other healthcare systems must align with the sites’ available resources, technical capacities (e.g., software choice, server type), and the UI standards (e.g., color schemes, button functions). This adaptation will require collaboration with IT teams and interface programmers. Ideally, institutions should allocate resources and efforts to present the tool to key clinic leaders and conduct usability testing to gain feedback using a UCD approach. For institutions with limited recourses, discount usability testing methods can be employed (Nielsen 1995; Brock et al. 2013). These may include think-aloud protocols with a small number of representative users, heuristic evaluations by experts, or simplified A/B testing (comparing version A to version B to see which is preferred by end users) of key interface elements. These methods can provide valuable insights at a fraction of the cost of full-scale usability studies. At a minimum, after validating the ML algorithm in their patient population, silent testing of the tool should be performed to ensure the tool is not burdensome on the users or leading to other unintended outcomes. After the planned pilot testing of our ML tool in a small number of UF primary care clinics, the results can inform future large-scale implementation of the tool in other UF Health clinics and non UF Health systems.

Our study and the design of our ML-driven opioid overdose CDS tool face several limitations and challenges. Firstly, the sample size of PCPs involved in the formative interview and usability sessions was relatively small, potentially limiting the representativeness of their opinions across UF Health primary care clinics and other settings. Second, our research highlights practical barriers and opportunities in implementing ML/AI-driven tools. For instance, leadership support facilitates CDS tool implementation, while coordinating across various teams, including IT and EPIC interface programmers, can be both time-consuming and costly. Finally, our project was limited to outpatient primary care settings. However, the insights and infrastructure derived could lay the groundwork for future deployments of similar tools in different healthcare environments, such as inpatient or emergency departments. Nevertheless, this initiative paves the way for novel AI technologies that healthcare professionals can use to enhance care for patients prescribed with opioids. It also addresses public concerns on AI tools by improving transparency in ML-driven CDS tools, thereby increasing trust in their usage.

## Conclusions

In summary, our approach in developing this ML-driven CDS tool for predicting opioid overdose in EHR systems underscores the importance of user-centered design and careful communication of risk during the integration of new technologies with existing healthcare systems to ensure effectiveness and mitigate bias.

## Supplementary Information

Supplementary Material 1.

## Data Availability

Due to the nature of the Institutional Review Board (IRB) approval and to protect study participants privacy, the transcripts and recordings from the formative interview sessions and usability sessions cannot be made publicly available.

## Abbreviations

AI: Artificial intelligence
API: Application programming interface
BPA: Best practice alert
CA: California
CDS: Clinical decision support
CTSI: Clinical and Translational Science Institute
EHR: Electronic health record
FAQ: Frequently asked questions
FHIR: Fast Healthcare Interoperability Resources
GAD-7: Generalized Anxiety Disorder Assessment
IDR: Integrated Data Repository
IT: Information technology
IRB: Institutional Review Board
MDQ: Mood Disorder Questionnaire
ML: Machine learning
MME: Morphine milligram equivalence
OIRD: Opioid-induced respiratory depression
ORT: Opioid Risk Tool
OUD: Opioid use disorder
PCORnet: Patient-Centered Outcomes Research Network
PCP: Primary care physicians
PDMP: Prescription Drug Monitoring Program
PHI: Patient health information
PHQ-9: Patient Health Questionnaire 9
RedCap: Research Electronic Data Capture
TN: Tennessee
UF: University of Florida
UF Health: University of Florida Health
UCD: User centered design
UI: User interface
US: United States
USA: United States of America
WA: Washington (state)
