# Supplementary material for: Design and development of a machine-learning-driven opioid overdose risk prediction tool integrated in electronic health records in primary care settings
Source: Bioelectron Med. 2024 Oct 18;10:24. doi: 10.1186/s42234-024-00156-3 (PMC11488086; doi:10.1186/s42234-024-00156-3)
Supplement: Supplementary file 1 — Supplementary Material 1. [file 42234_2024_156_MOESM1_ESM.docx]

**Supplemental Materials**

**Supplemental Table 1. Primary care physicians (n=4) feedback on the presentation of risk from the formative interviews on displaying risk**

**Supplemental Figure 1. User centered design approach used to develop the opioid overdose risk prediction clinical decision support (CDS) tool**

**Supplemental Figure 2. Before Input from end-users, UF Health information technology, and Integrated Data Repository Research Services: An illustration of a proposed workflow diagram for the opioid overdose risk prediction clinical decision support tool**

**Supplemental Table 1. Primary care physicians (n=4) feedback on the presentation of risk from the formative interviews on displaying risk**

| **Risk statement options** | **Primary care physicians feedback** |
| --- | --- |
| High risk captures 75-90% of patients who overdose | - This wording was most preferred. - This wording was not always understood. - The range was seen as being too large. - It was not clear where in the range the patient was. |
| This patient is in the top 5 percentile of overdose risk | - This wording was considered brief and digestible. - This wording was not always understood. |
| This patient has 10 times higher overdose risk than average | - This wording was viewed as convincing. - It was not clear what the risk level was higher than. |
| This patient has 2% chance of overdose | - This wording was not preferred by PCPs. |
| Combination of above options | - This wording was not preferred by PCPs. |


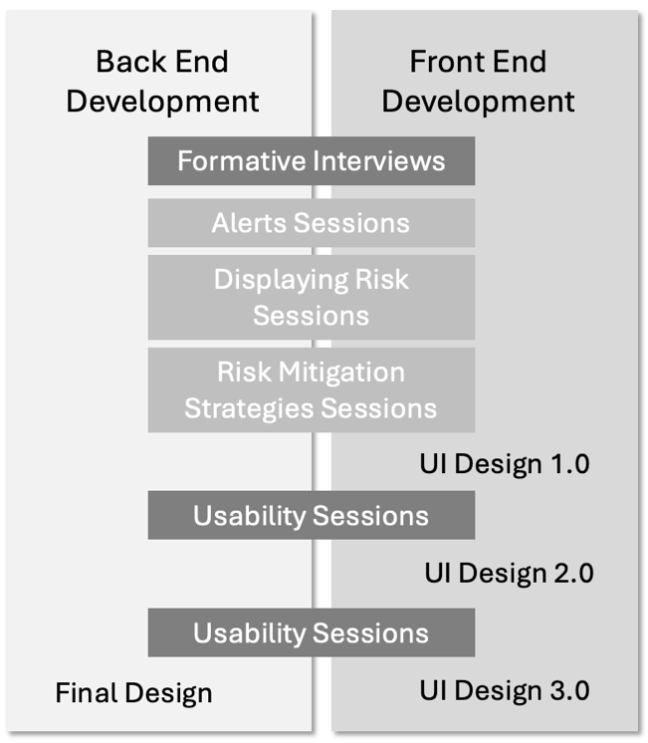


**Supplemental Figure 1. User centered design approach used to develop the opioid overdose risk prediction clinical decision support (CDS) tool**

Abbreviation: UI, user interface.


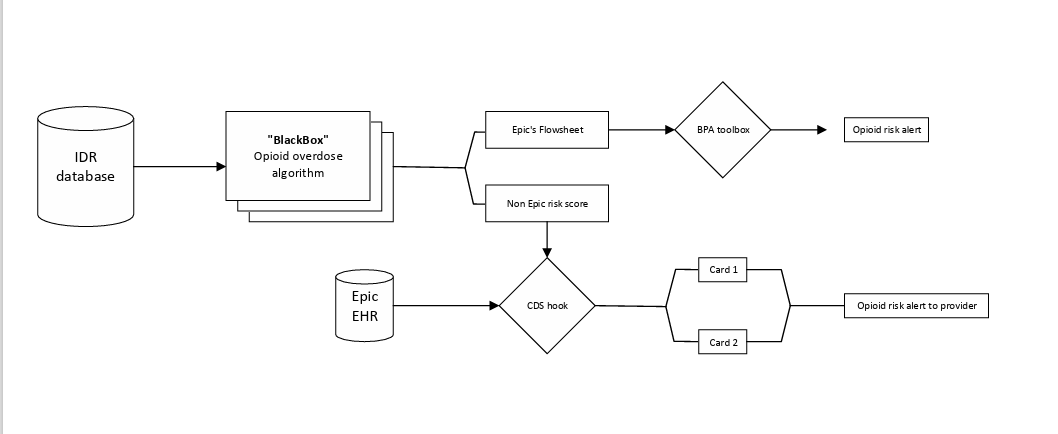


**Supplemental Figure 2. Before Input from end-users, UF Health information technology, and Integrated Data Repository Research Services: An illustration of a proposed workflow diagram for the opioid overdose risk prediction clinical decision support tool**

Abbreviations: BPA, best practice alert; EHR, electronic health records; IDR, Integrated Data Repository
